# Development of an In Vitro Method of Propagation for *Artemisia tridentata* subsp. *tridentata* to Support Genome Sequencing and Genotype-by-Environment Research

**DOI:** 10.3390/plants9121717

**Published:** 2020-12-05

**Authors:** Rachael Barron, Peggy Martinez, Marcelo Serpe, Sven Buerki

**Affiliations:** 1 Department of Biological Sciences, Boise State University, Boise, ID 83725, USA; rachaelbarron@u.boisestate.edu (R.B.); peggymartinez@boisestate.edu (P.M.); mserpe@boisestate.edu (M.S.); 2 Department of Plant Sciences, Simplot, Boise, ID 83706, USA

**Keywords:** drought, genotype, in vitro culture, plant growth regulators, sagebrush

## Abstract

Basin big sagebrush (*Artemisia tridentata* subsp. *tridentata*) is a keystone species of the sagebrush steppe, a widespread ecosystem of western North America threatened by climate change. The study’s goal was to develop an in vitro method of propagation for this taxon to support genome sequencing and genotype-by-environment research on drought tolerance. Such research may ultimately facilitate the reintroduction of big sagebrush in degraded habitats. Seedlings were generated from two diploid mother plants (2n = 2x = 18) collected in environments with contrasting precipitation regimes. The effects of IBA and NAA on rooting of shoot tips were tested on 45 individuals and 15 shoot tips per individual. Growth regulator and individual-seedling effects on percent rooting and roots per shoot tip were evaluated using statistical and clustering analyses. Furthermore, rooted shoot tips were transferred into new media to ascertain their continued growth in vitro. The results suggest that *A. tridentata* is an outbred species, as shown by individuals’ effect on rooting and growth. IBA addition was the most effective method for promoting adventitious rooting, especially in top-performing individuals. These individuals also have high survival and growth rates upon transferring to new media, making them suitable candidates for generating biomass for genome sequencing and producing clones for genotype-by-environment research.

## 1. Introduction

The unrelenting 21st-century megadrought in southwestern North America (SWNA) represents a major threat to ecosystems in the face of climate change [1]. Basin big sagebrush (*Artemisia tridentata* subsp. *tridentata*) is a keystone species of the sagebrush steppe, a widespread habitat within SWNA [2]. In addition to being ecologically dominant, this shrub has medicinal properties, valued historically by Native Americans, and is a required food resource for endemic and threatened pygmy rabbit and sage-grouse [3,4]. The sagebrush steppe was once distributed over more than 1 million km^2^, but has since been destroyed and fragmented due to invasive species, increased fire frequency, and habitat destruction [5]. As part of the GEM3 multi-disciplinary project, we seek to understand how genetic diversity and phenotypic plasticity affect basin big sagebrush response to environmental change, specifically drought, shaping both population response and adaptive capacity. Such genome-to-phenome research relies on controlled genotype-by-environment (GxE) experiments, where individuals representing different genotypes are exposed to contrasting treatments, and their phenotypic responses are measured. This approach relies on norms of reactions and statistical analyses to partition the importance of phenotypic plasticity vs. genomic processes underpinning the organism’s capacity to rapidly adapt to climate change. Such research also requires annotated genomes to ascertain the molecular mechanisms of adaptation. 

GxE experiments aim at disentangling phenotypic plasticity from genomic processes. Comparing inbred lines and clones’ responses to different treatments can markedly facilitate the disentangling of these processes [6,7]. *Artemisia tridentata* experiences outcrossing and has a generation time of approximately three years [8]. These characteristics and, in particular, the generation time make the production of inbred lines rather impractical. The other alternative is to use clones, but their production depends on developing an efficient vegetative propagation method. Such a method would represent a significant step towards genome-to-phenome research in *A. tridentata*. Furthermore, if the propagation were to be conducted in vitro, the clones would provide aseptic and genetically uniform plant material for whole-genome sequencing. A consideration in the generation of clones is selecting an approach that minimizes mutations [9,10]. In this regard, a method involving cultivating shoot segments, producing new shoots from their axillary buds, and iteration of these steps seems adequate to maintain genetic stability [11]. This approach does not depend on the formation of adventitious shoots, which, in tissue culture, can be a primary source of somaclonal variation [10,12]. 

Basin big sagebrush exists as a polyploid complex with both diploid (2n = 2x = 18) and tetraploid (2n = 4x = 36) cytotypes [13]. These cytotypes co-occur in the landscape, but the mechanism of polyploidization leading to tetraploid cytotypes remains mostly unknown [13,14]. Unlike individuals from the tetraploid cytotype, Richardson et al. [14] have shown, based on DNA sequencing, that all the sampled diploid individuals formed a monophyletic lineage, which exhibited limited genetic diversity. In this context, we have focused our efforts on developing in vitro propagation protocols for diploid basin big sagebrush. Our working hypothesis is that the limited genetic diversity observed in this monophyletic taxon (compared to the other lineages recovered in Richardson et al. [14]) should canalize the phenotypic response of the individuals included in our experimental design. To further reduce variability associated with genetic diversity, we developed a protocol based on half-siblings generated from the same mother plant. However, we included in the study two mother plants from environments that markedly differ in precipitation regimes and drought occurrences. Although not yet tested, we reasoned that individuals and clones derived from one mother plant might be more drought-resistant than those from the other. In other perennial species, GxE experiments using clones differing in drought tolerance have been valuable in identifying traits and genes that help plants cope with drought [15,16,17]. The availability of such clones in *A. tridentata* would allow us and other researchers to conduct similar studies. 

Efforts to clone big sagebrush in vitro have been limited. Turi et al. [18] have conducted in vitro conservation for *A. tridentata* (without specifying which ploidy level they focused on), but they could not initiate rooting from individual shoot tips. In this context, to our knowledge, only one study focusing on vegetative propagation from stem cuttings has been published on our focal species [19]. This study aimed at taking stem cuttings of several ecotypes to develop protocols to support post-fire restoration. The authors have shown that there was significant individual variation and that although synthetic auxin (IBA) was effective in promoting rooting, it could not overcome the individual effect [19]. The age of the plant and the timing of cuttings also significantly impacted the rooting of stem cuttings [19]. Although promising, this latter study called for developing an in vitro method of propagation using growth regulators on shoot tips from seedlings. Working in vitro might also allow the rapid establishment of individual lines for out-planting in GxE experiments or provide biomass for genome sequencing. Since the literature on in vitro methods of propagation for our focal species is limited, we summarize some key studies on other *Artemisia* species that have used non-woody shoot tips as their experimental material. We also report findings from a pilot study that we conducted on our focal species. These data enabled us to design the experiment presented here. The auxins indole-3-butyric acid (IBA), indole-3-acetic acid (IAA), and naphthalene acetic acid (NAA) have been used to induce adventitious root formation in regenerated shoot tips of other *Artemisia* species. A study on *A. vulgaris* found that the addition of 1.5 mg/L IAA resulted in a 98.2% root formation [20]. Alok et al. [21] reported that 1 mg/L IBA in the culture medium yielded the highest root formation in regenerated *A. pallens* shoots. An in vitro regeneration study in *A. annua* reported the highest root formation (85.8%) in regenerated shoots using 0.5 mg/L NAA [22]. Finally, our preliminary experiment has shown that diploid *A. tridentata* subsp. *tridentata* shoot tip cuttings can form adventitious roots in a medium lacking growth regulators [23]. However, the results suggested marked differences in adventitious root formation depending on the seedling from where the shoot tips originated; some individual seedlings produced shoot tips that either rooted at a very low frequency or not at all.

In the present study, we further characterized differences in adventitious rooting capacity among seedlings and investigated whether the addition of two synthetic auxins to the culture medium could lead to higher and more uniform rooting rates. We also wanted to ascertain that rooted shoot tips could survive and grow upon transferring into fresh medium since this is important to maintaining a clone and ultimately acclimate it to greenhouse and field conditions. Overall, this study aims at answering the following questions. Q1: Does the addition of growth regulators (here IBA and NAA) significantly increase rooting in diploid basin big sagebrush shoot tips? Q2: Are there some individuals more efficient at rooting, independent of treatment? If yes, those could be candidates to establish lines for genome sequencing and GxE experiments. Q3: Can we successfully transfer rooted shoot tips into fresh culture media and maintain seedlings in vitro? To answer the first two questions, we first harvested multiple shoot tips from individual seedlings grown in vitro. Subsequently, we distributed these shoot tips between treatments and plates following a scheme that allowed us to analyze the effect of the growth regulators on rooting and, more notably, whether the rooting rate was affected by the identity of the seedling from where the tips originated. For Q3, we tested whether the medium used for rooting and the number of roots per shoot tip affected their survival and growth upon transferring to medium without growth regulators. The results indicated that the addition of synthetic auxins to the culture media promotes rooting. However, statistical and clustering analyses also showed that the ability to form adventitious roots was influenced by intrinsic, yet not identified, characteristics of individual seedlings. In addition, the number of roots per shoot tip was an important factor in affecting subsequent growth in the medium lacking growth regulators.

## 2. Results

The protocols, data, and reproducible workflow (the R code, including citations and versions of all R packages) associated with this study are available on GitHub [24], and a companion GitHub Pages website was developed to fully explain our analyses [25].

### 2.1. Plant Materials

The plant material used in the study included 45 individuals, 23 G1 (from one mother plant from the ID3 population), and 22 G2 (from one mother plant from the UT2 population). For each individual (hereafter referred to as individual lines), 15 shoot tips were harvested. Each of these shoot tips was placed on a different plate to have three individual × growth-regulator treatment replicates (see Materials and Methods for detail). The list of all individuals and the raw data on callus and root development is available on the dedicated GitHub pages website [25].

### 2.2. Effect of Growth Regulators on in vitro Calli Development on Shoot Tips

**Figure 1:**
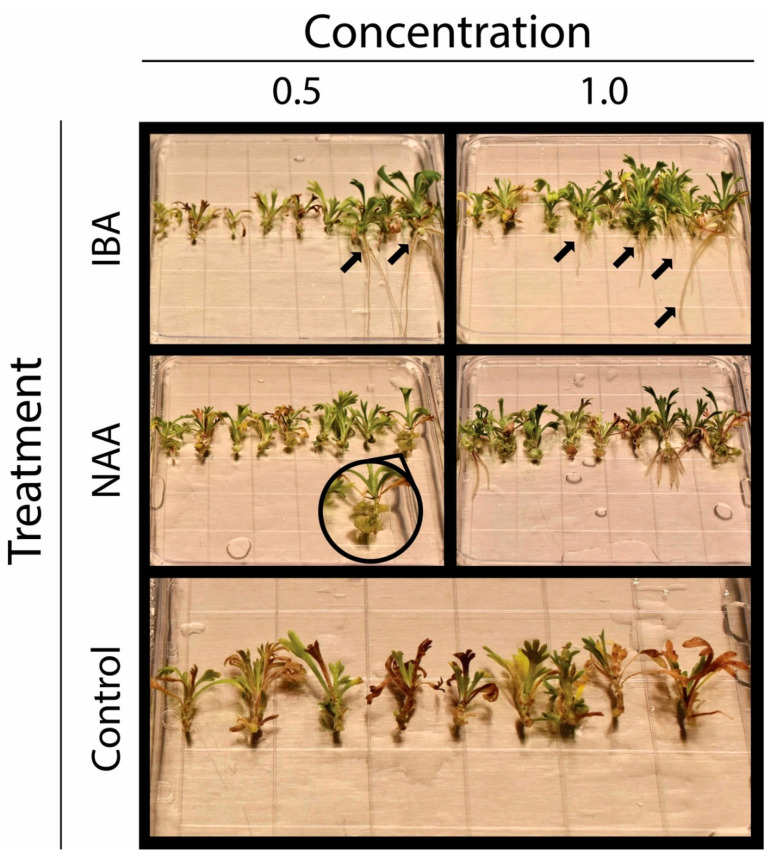
Representative observations of root (see arrows) and callus (see magnified zone) development in shoot tips of *Artemisia tridentata* subsp. *tridentata* sorted by growth-regulator treatment after 15 days in culture.

The effect of growth regulators on callus development is summarized in Table 1. Based on the GLM analysis, the number of shoot tips that formed callus was affected by treatment, but not by block. The Tukey’s tests on treatment showed that the only significant difference was between the control and any other growth-regulator treatment. Callus formation was minimal in the control medium without growth regulators (2.96%) and more than 75% in the medium with auxin (Figure 1).

**Figure 2:**
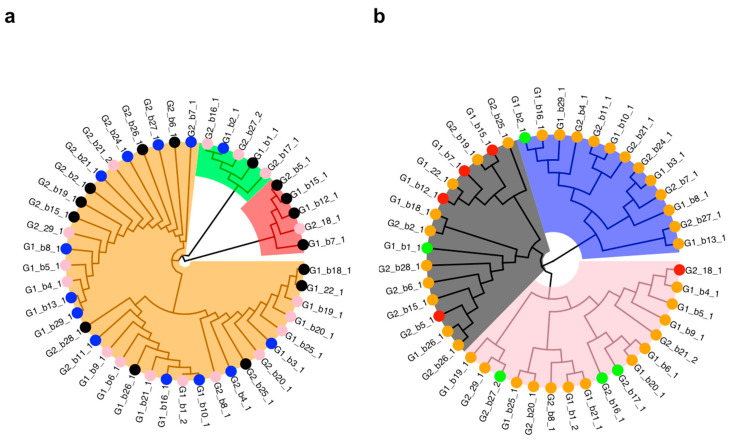
Clustering analyses based on callus (**a**) and root (**b**) data. For each variable, clusters are represented by shaded polygons, whereas the circles represent the assignment of the individual lines in the other analysis. Note, for contrast, the grey cluster in (**b**) is depicted with black dots in (**a**).

**Figure 3:**
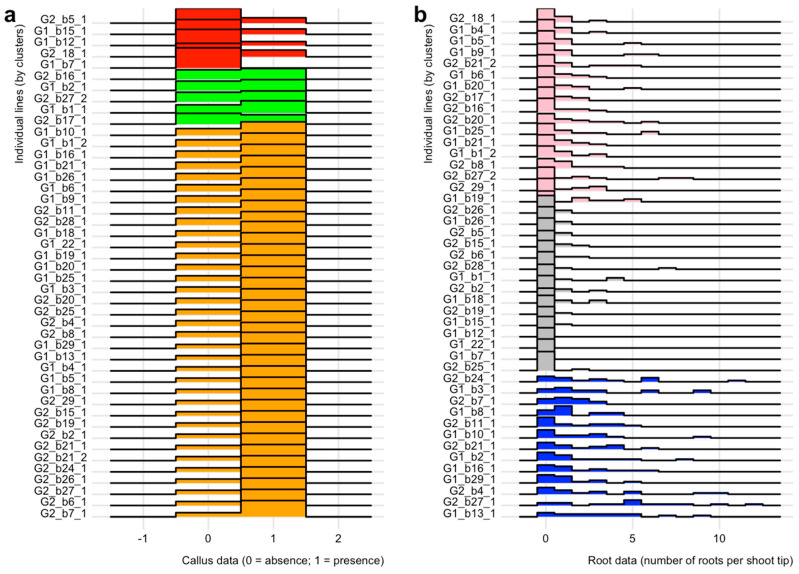
Ridgeline plots comparing callus (**a**) and root (**b**) formation in shoot tips of *Artemisia tridentata* subsp. *tridentata*. Clusters are identified by different colors (see Figure 2). On the y-axis, G1 or G2 refers to the genotype and the letters and numbers to individual lines.

The GLM analysis of genotype on callus development showed no difference between G1 and G2 (*p*-value = 0.4). However, there were differences between individual lines. The chi-square test of individual lines on callus formation had a *p*-value of 1.6 × 10^−6^. The results of the clustering analysis illustrate these differences (Figure 2 and Figure 3), which can be grouped into three clusters: (i) red cluster (*n* = 5 with 3 G1 and 2 G2): consisting of individual lines exhibiting no to minimal callus formation, (ii) green cluster (*n* = 5 with 2 G1 and 3 G2): consisting of individual lines with a frequency of callus formation between 47 to 60% and (iii) orange cluster (*n* = 35 with 18 G1 and 17 G2): consisting of individual lines with a callus formation frequency of 67 to 100%. Notably, none of the shoot tips from one individual line, G1_b7_1, formed callus, while the frequency of callus formation in any other individual line was at least 26.6%.

### 2.3. Effect of Growth Regulators on in vitro Rooting of Shoot Tips

Table 2 summarizes the effect of growth regulators on the rooting of shoot tips. The GLM analysis for the presence/absence of roots in shoot tips showed a significant treatment effect, but no block effect. As for callus, the Tukey’s test indicated that the only significant difference was between the control and any other growth-regulator treatment (Table 2). In treatments with a synthetic auxin, the percent of shoot tips with roots was at least four folds of the control. The other rooting response that we analyzed was the number of roots per shoot tip. The GLMNB analysis indicated both treatment and block effects. For treatment, the Tukey’s test revealed that IBA at 1 mg/L resulted in a higher number of roots per shoot tip than the NAA treatments, and all hormone additions enhanced rooting compared to the control (Table 2; Figure 1). The block’s effect on the number of roots per tip suggested differences between individual lines, since each block’s individual lines differed from those in any other block. These differences were investigated by a non-parametric test and clustering analysis (see below). 

As with callus, genotype did not affect the presence/absence of roots per tip (*p*-value = 0.87) or the number of roots (*p*-value = 0.78). However, there were differences between individual lines. The chi-square test for the individual lines’ presence/absence data yielded a *p*-value of 1.2 × 10^−15^. Similarly, the Kruskal–Wallis test for the individual line effect on roots per tip gave a *p*-value of 2.2 × 10^−16^. Further analysis focusing on the number of roots per tip was conducted using a Wilcoxon test to identify individual lines with the highest rooting capacity. This test revealed two individual lines, G2_b27_1 and G2_b7_1, that significantly (*p*-value < 0.01) outperformed at least 20% of the other lines.

The clustering analysis based on the number of roots per shoot tip (15 shoot tips in total) revealed three rooting clusters (Figure 2; Figure 3): (i) grey cluster (*n* = 15 with 7 G1 and 8 G2): comprising individual lines exhibiting no to very limited rooting, (ii) pink cluster (*n* = 17 with 9 G1 and 8 G2): comprising individual lines exhibiting limited rooting and (iii) blue cluster (*n* = 13 with 7 G1 and 6 G2): comprising individual lines showing high rooting capacities independent of treatment. As expected, the two top individual lines identified by the Wilcoxon test are in the blue rooting cluster (Figure 3). We also analyzed whether the clusters identified based on callus data matched those found for roots. In general, this comparison shows a lack of matching between the callus and root clusters (Figure 2). However, one trend in Figure 2 is that most blue cluster individual lines are within the callus orange cluster, which had the highest callus development frequency (Figure 2 and Figure 3).

The differences between rooting clusters could have been due to higher sensitivity to growth regulators in one cluster over the other or to differences in rooting capacity independent of treatment. To investigate these possibilities, we analyzed, by cluster, the effect of the growth regulator treatments on rooting (Table 3). This analysis suggested that differences between the grey and pink clusters were due to a difference in their sensitivity to the tested growth regulators. In both of these clusters, root formation in the control treatment was zero. The addition of growth regulators increased rooting, but this increase was more prominent in the pink than in the grey cluster (Table 3). The response to growth regulators between the pink and blue clusters was similar (not significant based on a *p*-value of 0.01). However, the blue cluster benefited from an apparent higher rooting capacity, independent of treatment; this notion is supported by the frequency of shoots forming roots in the control treatment, which was 30.8% (Table 3).

Independent of the reasons for the differences between rooting clusters, the analysis indicated that in all clusters, IBA, particularly at 1 mg/L, was the most effective treatment in promoting adventitious root formation. In the blue cluster, the IBA treatments also had a higher percent of shoots forming roots (87.2%) and a higher number of roots per shoot tip (ca. 3 roots per shoot tip) than any other cluster by treatment combination.

### 2.4. Survival and Plantlet Height of Rooted Shoot Tips Transplanted to Fresh Media

**Figure 4:**
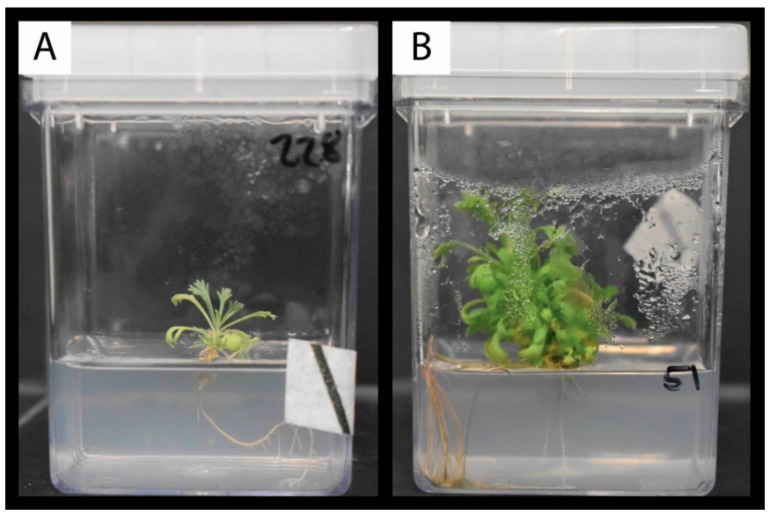
Visual comparison of plantlet heights after 5 weeks of culture. (**A**) Plantlet grown from the shoot tip of G1_b20_1 individual belonging to the pink rooting cluster (see Figure 2). (**B**) Plantlet grown from the shoot tip of G2_b27_1 individual belonging to the blue rooting cluster (Figure 2). Note: leaf shape differs from that of adult individuals due to the in vitro growing environment and heterophylly between juvenile and adult development phases.

A total of 42 individuals (G1:20/G2:22) representing 273 shoot tips (G1:138/G2:135) were transferred into GA-7 Magenta vessels containing 100 mL of MMS medium to monitor their survival and growth (Table 4; Figure 4). We transplanted a somewhat similar number of individuals per rooting clusters (with 12, 16 and 13 individuals for the grey, pink and blue rooting clusters). However, the number of rooted shoot tips per cluster markedly differed. Twenty-eight, 104, and 134 shoot tips were transferred into Magenta vessels for the grey, pink, and blue rooting clusters, respectively (Table 4). Furthermore, independent of the rooting clusters, none to very few shoot tips from the control treatment were included in this experiment, and all of these were from the blue cluster (Table 4). Three weeks after transplanting, the overall plantlets’ survival and mean height was 75.82% and 1.78 (± 1.21) cm, respectively. At five weeks, survival was 53.48%, and the mean height 1.69 (± 1.65) cm (see Figure 4).

**Figure 5:**
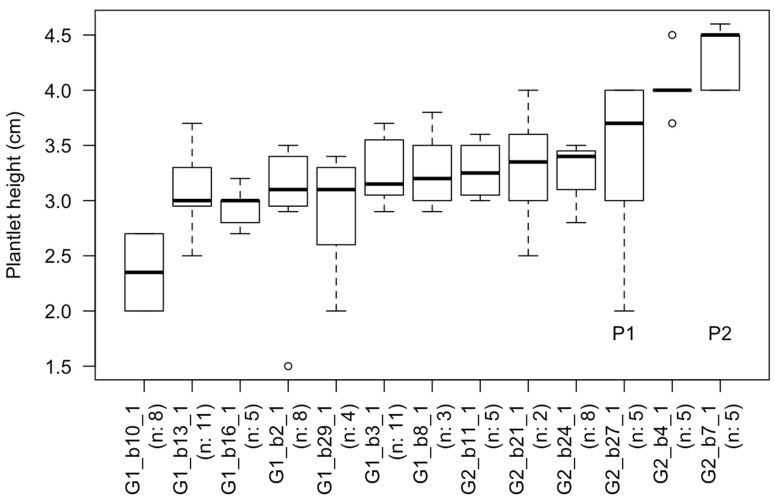
Boxplot showing plantlets heights after five weeks of culture for individuals belonging to the blue rooting cluster of *Artemisia tridentata* subsp. *tridentata*. The n indicates the number of plantlets cultured for each individual. The “P” indicates the top performers as identified by the statistical analyses.

Fisher exact tests indicated that the rooting cluster and media used to initiate rooting did not affect survival (*p*-values of 0.15 and 0.37 respectively). Although not significant, differences were observed between the NAA 0.5 mg/L and the IBA treatments; the rooted shoot tips transferred from the NAA 0.5 mg/L treatment had higher mortality than those transferred from media with IBA. The ANOVA analysis of plantlet heights yielded somewhat different results from those of survival, with rooting cluster showing a significant effect. Individual lines in the blue rooting cluster were significantly taller than those in the pink cluster (Table 4). In contrast, individual lines in the grey clusters were not different from those in the other clusters. This lack of difference may be due to the small sample size, since fewer shoot tips for the grey cluster were part of the experiment (Table 4). Within the blue cluster, the individual with the highest average height was G2_b27_1. This individual was significantly taller than 13.88% of the individual lines that survived the transplanting. The boxplot illustrates the distribution of plantlet heights after five weeks of culture for individuals in the blue rooting cluster (Figure 5). This boxplot also showed that the top two performers identified by the rooting experiment were among the tallest individuals (Figure 3b and Figure 5).

## 3. Discussion

### 3.1. Indirect Adventitious Rooting of Artemisia tridentata Shoot Tips

The initiation of adventitious roots in shoot explants may occur directly or indirectly [26]. Direct rooting involves the accumulation of endogenous auxin at the base of cuttings and subsequent differentiation of competent cells into root founder cells [27]. In contrast, in indirect rooting, a callus phase precedes root initiation [26]. The low percentage of shoot tips with roots in the control medium indicates that direct root formation was very limited in our explants. An exception may have been certain individual lines in the blue cluster, where 30% of the shoot tips formed roots without exogenous auxin (Table 3). Independent of this possibility, most shoot tips appeared to have developed roots indirectly. Based on our regular observations of the cultures, callus formation mostly came before root initiation. The synthetic auxin treatments also enhanced both callus and root formation, suggesting that callus development facilitated root initiation (Table 1 and Table 2, Figure 3). 

Culture media used to induce callus usually contains auxins and cytokinins [28,29]. In *A. tridentata* shoot tips, callus developed without cytokinins in the culture medium. While the main site of cytokinin synthesis is the root, synthesis of this hormone occurs throughout the plant and may increase with wounding [30,31]. Consequently, endogenous cytokinins, in combination with the exogenous auxins, may have contributed to callus development. Callus and subsequent root formation in media with just auxins are not uncommon [32]. However, one outstanding question is whether adding cytokinins could have resulted in a larger callus and potentially more roots per shoot tips. With this aim, a two-step procedure is sometimes used to attain indirect rooting; a medium with cytokinins and auxins first induces callus formation, and a second medium with only auxins promotes rooting [27]. This approach seems unnecessary for *A. tridentata* individual lines that showed several roots per shoot tip, but it may be worth testing in individual lines that only developed one or two roots (see Figure 2 and Figure 3). However, producing more callus is unlikely to entirely overcome the observed individual differences in rooting. As indicated by comparing callus and rooting clusters (Figure 2 and Figure 3), some individual lines with minimal root formation (grey rooting cluster) had a high percent of shoot tips with callus. Thus, even though they are morphologically similar, calli from different individual lines differed in their competence to produce roots.

### 3.2. Basin Big Sagebrush Exhibits High Individual Rooting Plasticity

Basin big sagebrush (*Artemisia tridentata* subsp. *tridentata*) is a polyploid complex, with diploid (2n = 2x = 18) and tetraploid (2n = 4x = 36) cytotypes, occurring across a broad ecological spectrum in western North America where hybridization has been extensively observed [2,8,13,14]. However, Richardson et al. [14] have shown that based on DNA sequencing, the diploid cytotype studied here was monophyletic and exhibited limited genetic diversity. We hypothesized that focusing on diploid individuals would canalize the phenotypic response and reduce individual effects on shoot tips’ rooting. To further lessen individual influences, we tested half-siblings grown from seeds collected from two mother plants growing in a contrasting climate and corresponding to our genotype hypotheses (G1: drought-tolerant, G2: drought-sensitive). Contrary to expectations, our statistical analyses indicated both individual and treatment effects, while differences in genotype did not impact rooting. These results suggest that the seeds’ provenance cannot predict the ability to produce adventitious roots, but rather that seedlings from the same mother plant exhibited high plasticity for this trait (see Figure 2 and Figure 3). The high individual variability agrees with the only other results on the adventitious rooting of this species [19], and it appears to indicate genetic variance for this trait. Although there is no clear genotype signal, the top-performing individual lines for rooting and growth all belonged to the G2 genotype (Figure 2, Figure 3 and Figure 5). However, the blue rooting cluster also included several individual lines from the G1 genotype (Figure 2 and Figure 3).

Before further delving into candidates for GxE research and generating biomass for genome sequencing, we are comparing our results to similar studies on other *Artemisia* species. Based on the material presented in the publications, no significant individual effects were discerned in *A. vulgaris* [20], *A. pallens* [21], and *A. annua* [22]. Unlike these latter species of *Artemisia*, which, to some extent, have undergone domestication processes, no breeding programs have been established for *A. tridentata* subsp. *tridentata*. These differences in the degree of domestication could have impacted their heterozygosity, resulting in lower heterozygosity and individual variation in the domesticated species than in basin big sagebrush.

As noted, the individuals in our study were half-siblings. The variation in adventitious rooting between half-siblings is intriguing. The growing environmental and developmental stage of the plants from where cuttings originate can affect their ability to form adventitious roots [33,34,35]. Differences in microclimate or competition between seedlings during in vitro growth from seeding to collecting shoot tips could have resulted in metabolic and physiological differences that affected rooting. Such conceivable effects make it difficult to determine the extent to which dissimilarities in rooting reflect differences in genetic or other factors. Nevertheless, if any, environmental differences in vitro were likely to be small (Figure 1 and Figure 4). Also, shoot tips were harvested from seedlings of the same age and all regions of each seedling. Thus, it seems unlikely that dissimilarities in environmental or developmental factors were responsible for the observed disparities in rooting competence. Based on these considerations, discrepancies in adventitious rooting between half-siblings appear to have a genetic basis, most likely due to cross-pollination and segregation in self-crosses [36]. Adventitious rooting is a quantitative genetic trait, and various studies have described differences in rooting competence within a species [37,38,39,40]. Our results are consistent with these observations, but they seem to go further by showing differences between half-siblings.

### 3.3. Growth Regulators Impact Rooting of Shoot Tips

Although this study demonstrated high individual phenotypic plasticity in adventitious rooting of shoot tips, it also ascertained that adding growth regulators promoted rooting. Statistical analyses showed that, overall, 1 mg/L IBA was the most efficient treatment promoting rooting (Table 2). Although the differences were not always significant with other growth-regulator treatments, 1 mg/L IBA also induced the highest percentage of shoot tips forming roots and the highest number of root per shoot tips on individual lines from the blue rooting cluster (Table 3). High concentrations of IBA also increased rooting in the other study on this species [19], but like in our experiment, IBA was not able to overcome individual variation. Among the other studies in *Artemisia* species, only Alok et al. [21] reported that the addition of 1 mg/L IBA resulted in the highest root formation frequency in regenerated *A. pallens* shoots. NAA at 0.5 mg/L was retrieved as the best treatment for *A. annua* [22]. This latter result is interesting since this treatment was shown to be statistically equal to 1 mg/L IBA for individual lines in the blue rooting cluster. Finally, the study on *A. vulgaris* found that the addition of 1.5 mg/L IAA yielded the highest root formation rate [20], but this growth regulator was not tested in our experiment.

### 3.4. Towards Selecting Individuals for in vitro Culture Propagation Program

Our comparative statistical analyses agreed in identifying 13 individual (seven G1 and six G2) belonging to the blue rooting cluster that are good candidates for establishing individual lines for GxE experiments and producing biomass for genome sequencing (Figure 2 and Figure 3). Among these individuals, two G2 individuals (G2_b27_1 and G2_b7_1) statistically outperformed at least 20% of the other individuals by producing more adventitious roots per shoot tip. Finally, only G2_b27_1 significantly outperformed 46.15% of the individuals. As judged by the plantlets’ height, individual lines in the blue rooting cluster (Figure 5) also grew faster upon transplanting to MMS medium than individuals in the pink rooting cluster. A rapid rate of elongation is a desired characteristic for obtaining a large number of clones. This task requires several iterations of collecting shoot tips, rooting them, and repeating this process. Alternatively, unrooted shoot tips could be treated to induce bud break and growth of axillary shoots; the latter are harvested, and these steps repeated until many clonal shoot tips are produced and used for rooting. In either case, a faster elongation rate reduces the time between iterations, increasing the speed of clonal propagation. Our statistical analyses showed that rooting cluster did not affect survival, which was about 50% for each cluster (Table 4). The reasons for mortality upon transplanting to new media are unclear, but they may be related to difficulties in inserting the adventitious roots in the new medium. In this regard, varying the gelling agent or its concentration may facilitate the establishment of rooted shoot tips upon transplanting. Overall, the high individual effect identified during rooting was carried over after transplanting, particularly for growth. In this context, we favor implementing an in vitro culture program based on the top two performers using IBA treatment to initiate rooting. This approach will allow us to produce the biomass necessary for genome sequencing. The 2C genome size of diploid *Artemisia tridentata* subsp. *tridentata* was estimated at 8.42 pg, resulting in a diploid genome of 8.2 Gbp [41,42]. Based on our estimations, we will need >200 g of fresh biomass (leaves) to perform the DNA extractions required to sequence this large genome at 100× (using mostly PacBio technology complemented with Illumina reads) and confidently assemble it. For comparison, the 2C genome size of *Artemisia annua*, a sister species with the same chromosome number as our focal species, is estimated at 3.5 pg, corresponding to a diploid genome of 3.4 Gbp [42]. The haploid draft genome of *A. annua* was recently published and showed a high heterozygosity level (ca. 1.5%), which complicated genome assembly [43]. Based on the data presented here, we hypothesize that the 2.5× bigger genome size of *A. tridentata* subsp. *tridentata* and outbreeding in this species will also result in high heterozygosity, therefore calling for the need to pursue our in vitro culture program to establish lines of known genomes.

### 3.5. Perspectives and Future Work

Through this study, we developed an in vitro rooting procedure for big-basin sagebrush as a first step to obtain clones for genome sequencing and GxE experiments. The comparison between individuals suggests that G2_b27_1 is a good candidate to generate the biomass required for genome sequencing. The use of clones in GxE experiments will require implementing other protocols to transfer plantlets to soil and acclimatize them to greenhouse or field conditions. The acclimated clones will play a key role in assessing the proportion of phenotypic plasticity vs. genomic process underpinning rapid response to drought and climate change in general. In this regard, it would be important to increase the number of cloned individuals to obtain a set of clones that better represents the field’s genotypes. Apart from their uses in genome sequencing and GxE experiments, in vitro rooting could also have other applications. In their study, Alvarez-Cordero and McKell [19] stressed the importance of in vitro propagation of pre-adapted individuals to facilitate the post-fire restoration of the sagebrush steppe. Our research contributes to such a goal.

## 4. Materials and Methods 

### 4.1. Plant Materials

Seeds from the two mother plants of diploid *Artemisia tridentata* subsp. *tridentata* (2n = 2x = 18) used for this study were provided by the US Forest service and collected from two source-populations in Idaho (ID3: Latitude 43.336, Longitude −116.964) and Utah (UT2: Latitude 38.306, Longitude −109.387) in 2009 [44]. The taxonomy of this species is still debated, and we apply here the taxonomic concept used in Richardson et al. [14] and Chaney et al. [44]. Mother plants were identified using morphological features together with phylogenetic analyses [14] and flow cytometry [14,44]. We chose these two populations because they grow in sites with distinct precipitation regimes [44]. The plants growing in ID3 received ten times less rainfall than those in UT2. As a result, the first location experienced severe summer droughts, whereas the other did not due to the onset of the North American Monsoon in August [44]. Although not formally tested yet, these data suggest that the ID3 population may contain more drought-tolerant individuals than the UT2 population. Hereafter, half-siblings originating from these populations will be referred to as G1 (ID3, drought-tolerant) and G2 (UT2, drought-sensitive) genotypes (see below). The seedlings from one mother plant are denoted as half-siblings, because outcrossing occurs in *A. tridentata*, and the copious production of pollen suggests that cross-fertilization is common [8,44,45,46]. However, to our knowledge, the extent of outcrossing has not been well characterized, and is likely to vary with plant density and environmental conditions. Based on these considerations, the seeds used most probably originated from both self- and cross-fertilization.

Seeds (*n* = 200) from one mother plant (i.e. half-siblings) per population were counted and surface-sterilized. For this purpose, seeds were first rinsed in running water for 2 h and then incubated for 10 min in a 0.5% (*v/v*) sodium hypochlorite solution containing a surfactant (0.1% TritonX-100). Subsequently, the seeds were rinsed four times with sterile water for 5 min per rinse. Following surface sterilization, six to seven seeds were transferred to GA-7 Magenta vessels (Sigma-Aldrich, Saint Louis, MO, USA, V8505) onto 100 mL of growth medium. This medium (hereafter referred to as MMS for modified Murashige and Skoog medium) contained ½ strength MS micro and macronutrients [47], ½ strength modified Gamborg (B5) vitamins [48], 1% (*w/v*) sucrose, 3 g/L phytagel, and 1 mL/L PPM (Plant Preservative Mixture, Plant Cell Technology). Before autoclaving, the pH of the MMS medium was adjusted to 5.8. After seeding, vessels were placed into a growth chamber (Percival, model CU41L4, Perry, IA, USA) and kept under constant 20 °C temperature with a 16 h photoperiod, during which LED lamps supplied approximately 300 µmol m^−2^ s^−1^ of PAR. Fourteen days post-planting, 94% of G1 genotype and 99% of G2 genotype seeds had germinated. Seedlings were grown for 180 days before harvesting shoot tips for the experiment.

### 4.2. Experimental Media Composition and Explant Preparation

The rooting of shoot tips was tested in five media: MMS lacking growth regulators (control treatment), MMS with 0.5 mg/L IBA (Indole-3-butyric acid, Sigma-Aldrich, Saint Louis, MO, USA, CAS 133-32-4), MMS with 1.0 mg/L IBA, MMS with 0.5 mg/L NAA (Naphthalene acetic acid, Sigma-Aldrich, Saint Louis, MO, USA, CAS 86-87-3), and MMS with 1.0 mg/L NAA. Both synthetic auxins were filter-sterilized and added to the autoclaved MMS medium. For the experiment, we prepared 15 (12 × 12 cm) square Petri plates for each medium and harvested fifteen shoot tips (~1cm) from each of 45 individual seedlings (hereafter referred to as individual lines). Before placing the shoot tips on plates, we randomly divided the individual lines into five blocks, each with a different set of nine individual lines. Every block had 135 shoot tips: 5 treatments × 3 plates per treatment × 1 shoot tip from each of nine individual lines per plate. Thus, the experiment had three replicates per treatment × individual line combination and a total of 675 shoot tips (5 blocks × 135 tips per block). After transferring the shoot tips, the plates were sealed with parafilm and pierced with a few holes to allow airflow. The plates were positioned at a 45° angle in a growth chamber kept at 23 °C with a 16 h photoperiod. After two weeks of incubation, the presence/absence of calli and the presence and number of roots per shoot tip were scored and used for statistical and clustering analyses (see below).

### 4.3. Comparative Analyses on In Vitro Culture Data

The effect of growth regulators was assessed on three response variables: the number of shoot tips per plate that formed callus, those that developed roots (presence/absence), and the total number of roots. The latter served as a proxy for the number of roots per shoot tip. The presence/absence of callus and roots was evaluated using a generalized linear model (GLM) with Poisson distributed errors. The treatments’ impact on the total number of roots was analyzed using a negative binomial generalized linear model (GLMNB). The deviance goodness of fit test ascertained models’ fitness, as judged by *p*-values above 0.05. Post-hoc multiple comparisons were analyzed with Tukey’s significant difference test. The GLM, GLMNB, and post-hoc analyses were conducted in R [49] using the glm, glm.nb, and emmeans functions, respectively (see 25 for a list of used R packages together with their citations).

We examined the effect of genotype on the three response variables using each individual line as a replicate, thus resulting in 23 and 22 replicates for G1 and G2, respectively. The effect of genotype on callus was analyzed with a GLM with a Poisson distribution. Possible differences between genotypes on rooting (binary and number of roots per shoot tip) were examined using a GLMNB model. The fitness of the models was tested as described earlier.

Differences between individual lines in their ability to form callus and roots were evaluated using non-parametric tests and clustering analyses. These analyses were conducted independently of treatment due to the small number of replicates for each individual line x treatment combination. Binary data were analyzed by Chi-square [49]. A Wilcoxon rank-sum test was used to compare the number of roots per shoot tip [49]. With this test, the aim was to identify the individual lines with higher rooting capacity (top-performing individual lines). The top-performers were selected based on the criterion that they significantly (adjusted *p*-value < 0.01) outperformed at least 20% of the other individual lines. Callus and rooting (number of roots per shoot tip) data were also examined by clustering analyses. These analyses were performed between individual lines based on the Euclidean distance and the hierarchical clustering method implemented in the R *stats* package [49]. The callus clusters were mapped onto the root clusters to ascertain whether the former were congruent with the latter. To further compare the individual lines within each cluster, ridgeline plots were inferred for the callus and rooting using the R package *ggridges* [50].

### 4.4. Survival and Plantlet Height of Rooted Shoot Tips Transplanted to Fresh Media

After eighteen days from the day they were cut and placed in square plates, rooted shoot tips were transferred into GA-7 Magenta vessels containing 100 mL of MMS medium lacking growth regulators. The aim was to ascertain that they could continue growing in culture. Survival and growth of the rooted explants were measured three and five weeks after transfer to the GA-7 vessels. Growth was estimated by measuring the height of the seedlings. Survival data collected after five weeks were analyzed using the Fisher exact test [49]. The effects of treatment, rooting cluster, individual line on height were analyzed using ANOVA after ascertaining the normality and variance homogeneity of the data by the Shapiro–Wilk and Levene’s test, respectively [49]. Post-hoc tests were performed using the *emmeans* package in R [49], with *p*-values adjusted by the Tukey method. A boxplot comparing the distribution of plantlet heights for individuals belonging to the best rooting cluster was performed using the base R *boxplot* function [49].

## Figures and Tables

**Table 1:** Effect of growth regulators on in vitro callus development of shoot tips of *Artemisia tridentata* subsp. *tridentata*. Response based on the absence or presence of callus in each shoot tip. Values represent the mean ± SE of 15 plates with nine tips per plate. Values followed by different letters are significantly different (*p*-value < 0.01) based on Tukey’s test.

Growth Regulator	Concentration (mg/L)	Response (%)
Control	-	2.9 ± 1.5 ^b^
IBA	0.5	75.5 ± 7.5 ^a^
IBA	1	84.4 ± 7.9 ^a^
NAA	0.5	81.5 ± 7.8 ^a^
NAA	1	87.4 ± 8.04 ^a^

**Table 2:** Effect of growth regulators on in vitro rooting of *Artemisia tridentata* subsp. *tridentata* shoot tips. The rooting response was evaluated based on the presence/absence of roots (response %) and the average number of roots per shoot tip (simplified as Av. No. of Roots in the table header). Values represent the mean ± SE of 15 plates with nine tips per plate. Within a column, values followed by different letters are significantly different (*p*-value < 0.01) based on Tukey’s test.

Growth Regulator	Concentration (mg/L)	Response (%)	Av. No. of Roots
Control	-	8.9 ± 2.5 ^b^	0.13 ± 0.04 ^c^
IBA	0.5	47.4 ± 5.9 ^a^	1.37 ± 0.20 ^ab^
IBA	1	60.0 ± 6.6 ^a^	1.92± 0.26 ^a^
NAA	0.5	40.7 ± 5.5 ^a^	0.74 ± 0.12 ^b^
NAA	1	40.0 ± 5.4 ^a^	1.00 ± 0.15 ^b^

**Table 3:** Effect of growth regulators on in vitro rooting of *Artemisia tridentata* subsp. *tridentata* shoot tips sorted by rooting clusters. The rooting response was evaluated based on the presence/absence of roots (response %) and the average number of roots per shoot tip (simplified as Av. No. of Roots in the table header). Values represent the mean ± standard errors of 13 to 17 individual lines with three tips per individual line × treatment combination.

Cluster	Growth Regulator	Concentration (mg/L)	Response (%)	Av. No. of Roots
grey	Control	-	0 ± 0	0 ± 0
grey	IBA	0.5	11.1 ± 4.2	0.22 ± 0.10
grey	IBA	1	20.0 ± 7.1	0.44 ± 0.21
grey	NAA	0.5	13.3 ± 6.3	0.16 ± 0.08
grey	NAA	1	13.3 ± 5.4	0.22 ± 0.13
pink	Control	-	0 ± 0	0 ± 0
pink	IBA	0.5	49.0 ± 8.1	1.16 ± 0.19
pink	IBA	1	74.5 ± 6.1	1.82 ± 0.32
pink	NAA	0.5	39.2 ± 6.5	0.63 ± 0.12
pink	NAA	1	43.1 ± 4.7	0.86 ± 0.2
blue	Control	-	30.8 ± 8.8	0.62 ± 0.23
blue	IBA	0.5	87.2 ± 4.7	2.97 ± 0.37
blue	IBA	1	87.2 ± 6.0	3.59 ± 0.45
blue	NAA	0.5	74.4 ± 9.4	1.74 ± 0.37
blue	NAA	1	66.7 ± 6.5	2.28 ± 0.33

**Table 4:** Survival and plant height (cm) of 5-week-old plantlets of *Artemisia tridentata* subsp. *tridentata* transferred into Magenta vessels with MMS medium without growth regulators sorted by rooting cluster and the media used to initiate rooting (growth regulator treatment in Section 2.2 and Section 2.3). The total number of individual lines and shoot tips per rooting cluster indicates the variation in sample size.

Cluster	N. Individual Lines	N. Shoot Tips	Growth Regulator	Concentration (mg/L)	Survival (%)	Height (cm)
grey	0	0	Control	-	NA	NA
grey	5	5	IBA	0.5	40.00	1.2+/−1.64
grey	4	6	IBA	1	33.33	0.9+/−1.59
grey	4	6	NAA	0.5	66.67	2.27+/−1.94
grey	8	10	NAA	1	40.00	1.32+/−1.71
TOTAL	12	27				
pink	0	0	Control	-	NA	NA
pink	14	24	IBA	0.5	37.50	1.04+/−1.39
pink	16	35	IBA	1	60.00	1.82+/−1.59
pink	13	19	NAA	0.5	42.11	1.28+/−1.59
pink	15	26	NAA	1	53.85	1.56+/−1.57
TOTAL	16	104				
blue	8	12	Control	-	58.33	1.97+/−1.8
blue	13	32	IBA	0.5	65.62	2.27+/−1.7
blue	13	34	IBA	1	64.71	1.95+/−1.54
blue	12	27	NAA	0.5	37.04	1.21+/−1.64
blue	13	29	NAA	1	68.97	2.29+/−1.64
TOTAL	13	134				
